# Changes in Gray Matter Induced by Learning—Revisited

**DOI:** 10.1371/journal.pone.0002669

**Published:** 2008-07-23

**Authors:** Joenna Driemeyer, Janina Boyke, Christian Gaser, Christian Büchel, Arne May

**Affiliations:** 1 Department of Systems Neuroscience, University of Hamburg (UKE), Hamburg, Germany; 2 Department of Psychiatry, University of Jena, Jena, Germany; Baylor College of Medicine, United States of America

## Abstract

**Background:**

Recently, activation-dependant structural brain plasticity in humans has been demonstrated in adults after three months of training a visio-motor skill. Learning three-ball cascade juggling was associated with a transient and highly selective increase in brain gray matter in the occipito-temporal cortex comprising the motion sensitive area hMT/V5 bilaterally. However, the exact time-scale of usage-dependant structural changes occur is still unknown. A better understanding of the temporal parameters may help to elucidate to what extent this type of cortical plasticity contributes to fast adapting cortical processes that may be relevant to learning.

**Principal Findings:**

Using a 3 Tesla scanner and monitoring whole brain structure we repeated and extended our original study in 20 healthy adult volunteers, focussing on the temporal aspects of the structural changes and investigated whether these changes are performance or exercise dependant. The data confirmed our earlier observation using a mean effects analysis and in addition showed that learning to juggle can alter gray matter in the occipito-temporal cortex as early as after 7 days of training. Neither performance nor exercise alone could explain these changes.

**Conclusion:**

We suggest that the qualitative change (i.e. learning of a new task) is more critical for the brain to change its structure than continued training of an already-learned task.

## Introduction

While traditional research has focussed on functional forms of neuroplasticity, current theoretically based concepts suggest that structural cortical plasticity in adult brains plays a crucial role in adaptation to environmental changes and disease. Support for this hypothesis comes from studies demonstrating activity-dependent selective changes in gray matter induced in human adults [1]–[4]. However, these studies either used skill as a parameter and did not include a time pattern at all [3]; [4] or, in the case of longitudinal studies [1], investigated the exercise dependant changes only in 3 month intervals without controlling for how long or how intensely the volunteers practiced. Therfore, the exact time-scale at which such usage-dependant structural changes occur is still unknown [5].

We were therefore interested in the temporal details of structural neuroplasticity, as this knowledge may help to elucidate to what extent this type of cortical plasticity is involved in mediating short- and long-term clinical effects. Focussing on this issue, we replicated the previously used longitudinal study design [1] in 20 healthy young volunteers. Volunteers were investigated before and after 1, 2 and 5 weeks after beginning to juggle, controlling the amount of daily practice. It needs to be mentioned that we were not able to control against or quantify mental rehearsal (conscious or unconscious), which may have just as much impact on cortical reorganization as the actual juggling. We then asked the volunteers to stop exercising and scanned again after 2 and after 4 months. We predicted that learning three-ball cascade juggling will induce a transient and highly selective change in occipito-temporal areas as early as within the first two weeks.

## Materials and Methods

### Volunteers

We studied 20 healthy volunteers (11 female, 9 male; mean age 26.5 yrs). None of the volunteers was able to juggle before entering the experiment and none suffered from any diseases. Volunteers were recruited locally and they were informed that the purpose of the current study was to investigate the central nervous system's adaptive behavior to learning to juggle. The study was given ethical approval by the local Ethics committee (Ärztekammer Hamburg) and written informed consent was obtained from all study participants prior to examination.

### VBM-data acquisition

All volunteers received six T1-weighted MRI scans. The first scan was performed at the start of the study. Then all volunteers received 3 juggling balls and were instructed on how to learn a 3 ball cascade. The second scan was performed after 7 days, when volunteers demonstrated skilled performance (at least 60 seconds of endurance juggling), tested by one of the authors. A third and fourth scan were carried out another 7 and 28 days later, when the volunteers were asked to demonstrate at least 120 seconds and 180 seconds respectively, of endurance juggling. After the fourth scan, none of the “jugglers” was allowed to practice their juggling skills further. For most “jugglers” the three-ball cascade juggling at the time of the last two scans (scan 5 after two and scan 6 after four months) was still fluent, however significantly less than at time points 2–4 (again tested by one of the authors).

Magnetic resonance imaging (MRI) was performed on a 3T MRI system (Siemens Trio) with a standard headcoil. For each time point, a T1 weighted structural MRI was acquired for each subject using a 3D-FLASH sequence (TR 15 ms, TE 4.9 ms, flip angle 25°, 1 mm slices, FOV 256×256). T1 MR-imaging showed no morphological abnormalities or artefacts.

### VBM protocol

Data pre-processing and analysis were performed with SPM2 (Welcome Department of Cognitive Neurology, London, UK) running under Matlab (Mathworks, Sherborn, MA, USA) and described in detail elsewhere [1]; [6]. In short, pre-processing involved coregistration, spatial normalization, gray matter segmentation and 10 mm spatial smoothing with a Gaussian kernel. For the pre-processing steps, we registered all scans of each subject to the first scan to remove positional differences between the scans of each individual. The parameters for the following spatial normalization to the template were estimated using the first scan of each individual and were applied to the scans from all time-points. To facilitate an optimal segmentation, we estimated normalization parameters while removing non-brain voxels (skull, sinus) using a previously described optimized protocol [7] and a scanner- and study-specific gray matter template. The optimized parameters, estimated while normalizing extracted GM images to the customized GM template, were reapplied to the original whole brain images. The images aligned with the stereotactic space defined by the Montreal Neurological Institute (MNI) [8], were corrected for non-uniformities in signal intensity and partitioned into gray (GM) and white matter (WM) and cerebrospinal fluid and background (CSF) using a modified mixture model cluster analysis. Subsequently, all segmented unmodulated images were smoothed by convolving them with an isotropic Gaussian kernel of 10 mm full-width at half maximum (FWHM).

### Statistical analysis

The first analysis was a longitudinal analysis using a voxel by voxel paired t-statistic in order to detect regional differences in gray matter over all six time points. To confirm the findings of our previously data set, we tested for any regions that showed a transient increase of gray matter values during the training period. In fact, we tested whether the values of time point 2–4 (training period) were larger compared to the first time point (before the learning period) and to the time points five and six (after training had stopped). Additionally, we tested also for an increase at the second time point (after one week training) compared to the first time point (before training) to explore whether we are able to find a learning effect even after one week training. No other time courses were tested. Because we had a strong a priori hypothesis (hMT/V5 [1]), we applied a threshold of p<0.001 (uncorrected) across the whole brain.

The second analysis was a mean effect analysis (allowing us to combine data from different scanners [6]) of all data sets from our previously published cohort (n = 12, 3 time points, 1.5T Siemens scanner) [1] and the present data set (n = 20, 3 time points, 3T Siemens scanner). As the previous data set tested for an increase between the first two time-points (before the learning period in relation to the time of skilled performance) followed by a decrease to the last time-point (after training had stopped), we chose time point 1, 4 and 6 of our present study to test for medium effects in both groups (no conjunction.) We applied a threshold of p<0.001 (uncorrected) across the whole brain.

Additionally, we performed a regression analyses using performance and exercise (hours per day) as covariates to test for possible correlations. Again, we applied a threshold of p<0.001 (uncorrected).

## Results

### Longitudinal analysis

Including all time points, we found an increase in the middle temporal area of the visual cortex (hMT/V5) on the right (x = 33, y = −87, z = 0; Z = 4.56; p<0.001 uncorrected) and the left side (x = −29, y = −89, z = 2; Z = 3.25; p<0.001 uncorrected) for the time-points of skilled performance (scans 2–4) compared to time point one (scan 1). This pattern reversed when study participants were examined at time points 4 and 5 (scans 4 & 5 following the weeks of exercise).

**Figure 1:**
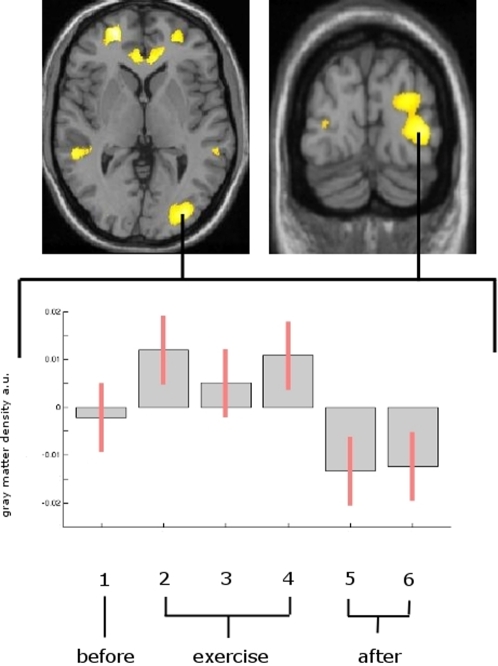
Transient structural changes superimposed on a normalized T1-image. Gray matter increase is shown superimposed on a normalized T1-image. The left side of the picture is the left side of the brain. a.u. = arbitrary units. Figure 1 top: Statistical parametric maps demonstrating the transient structural changes during the time of skilled performance (scans 2–4) compared to time point 1. A significant gray matter increase was found in the midtemporal area (hMT/V5) and in the frontal and temporal lobes and the cingulate cortex bilaterally. This pattern reversed when study participants were examined at time points 5 and 6 (following the weeks of exercise). Figure 1 bottom: mean and 90% confidence interval of the voxels of maximum intensity (right hMT) representing the gray matter expansion over time. Each box represents one scan (scan 1 =  before training, scans 2–4 = 7, 14 and 21 days after scan one and during the exercise period; scan 5 after two and scan 6 after four months (after training had stopped.)

**Figure 2:**
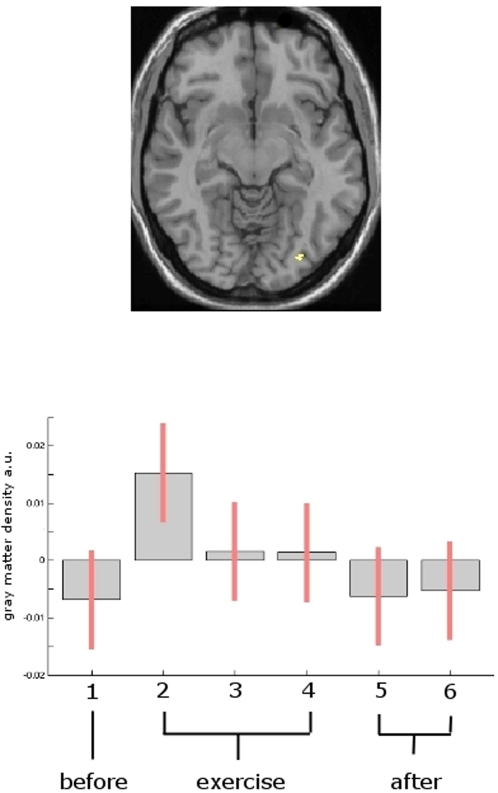
Statistical parametric maps demonstrating structural changes after 7 days. Figure 2 top: Statistical parametric maps demonstrating the transient structural changes after 7 days compared to time point one. A gray matter expansion between the first and the second scan was found in the midtemporal area (hMT/V5) on the right side, demonstrating that learning to juggle can change the gray matter in hMT/V5 as early as after 7 days of training. Note, that this change is a trend only (p<0.005, uncorrected). Figure 2 bottom: Box plot of the voxels of maximum intensity (right hMT/V5) representing the gray matter expansion over time.

Additionally, we found a significant change (p<0.001, uncorrected) of brain gray matter which followed the same time pattern (increase during exercise which receded when exercise stopped) bilaterally in the frontal and temporal lobes and the cingulate cortex (Table 1 and Figure 1). The changes in the occipital area were already detectable after one week of exercise. (Figure 2)

**Table 1:** Significant changes of gray matter

		*Transient increase in gray matter during exercise (n = 20)*			
Region	Brodman areas	Talairach coordinates			Z score of peak activation
		x	y	z	
hMT/V5 R	18	33	−87	0	Z = 4.56
hMT/V5 L	18	−29	−89	2	Z = 3.25
Inferior parietal lobule	40	0	−45	40	Z = 4.64
Superior frontal gyrus R	10	25	52	−2	Z = 4.06
Superior frontal gyrus L	10	−23	54	1	Z = 5.09
Medial temporal gyrus R	21	57	−33	−4	Z = 4.55
Medial temporal gyrus L	21	−56	−40	0	Z = 4.22
Cingulate cortex R	24	17	39	10	Z = 4.46
Cingulate cortex L	24	−5	35	−1	Z = 4.28
		***Mean effect analysis (n = 32)***			
hMT/V5 L	18	−38	−82	4	Z = 3.12
hMT/V5 R	18	41	−80	2	Z = 4.06

Significant changes (increase of cerebral gray matter) during the time of skilled performance (scans 2) compared to time point one (before juggling). This pattern reversed when study participants were examined at time points 5 and 6 (following the weeks of exercise). The changes are tabulated in terms of the brain region and the corresponding Brodmann's area (BA). The x, y, z co-ordinates are according to the MNI atlas. Each location is the peak within a cluster (defined as the voxel with the highest Z-score).

L = left, R = right,

### Mean effect analysis

**Figure 3:**
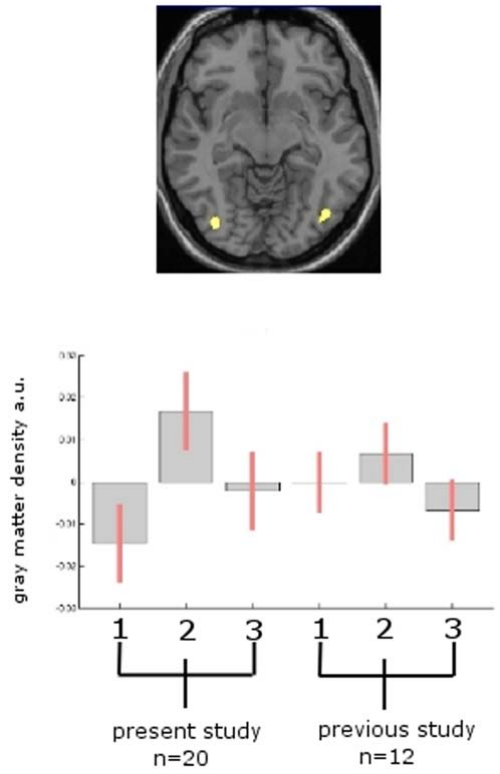
Mean effects analysis. Figure 3 top: The mean effect analysis of the previous data set (1.5 Tesla) of 12 volunteers [1] and the present data set (3 Tesla) of 20 volunteers showed that both cohorts exhibit transient gray matter increase in the hMT/V5 bilaterally (right: x = 41, y = −82, z = 4; Z = 4.06; left: x = −38, y = −80, z = 2; Z = 3.12). Figure 3 bottom: Box plot of the parameter estimates for both samples in the MT-area (right hMT/V5) using the contrasts described in the text to test for medium effects in both groups (no conjunction). The cluster is displayed with p<0.001 (uncorr.) and all parameter estimates (betas) in this cluster were averaged.

The mean effect analysis of the previous data set of 12 volunteers [1] and the present data set of 20 volunteers showed that both cohorts exhibit transient gray matter increase in the V5/hMT bilaterally (right: x = 41, y = −82, z = 4; Z = 4.06; left: x = −38, y = −80, z = 2; Z = 3.12) (Figure 3).

### Regression analysis

Testing for a correlation between exercise or performance and changes in gray matter produced no significant results.

## Discussion

Using the same paradigm, we are able to confirm and extend our previous finding of transient training-induced gray matter changes in the adult human brain. Our results show that dynamic alterations in gray matter structure can occur very rapidly within a time range of a single week (Figue 2).

This time-course favours fast adjusting neuronal systems, such as spine and synapse turnover [9] as the underlying factor for gray matter increase, rather than such slow evolving mechanisms as neuronal or glial cell genesis [10]. It is important to mention, that gray matter does not necessarily mean that we are measuring neurons or even cells as such. It is possible that other factors could subtly alter voxel values resulting in tissue being “misclassified” as gray matter. In general, an increase in gray matter could be due to an increase in cell size, neural or glial cell genesis, spine density or even changes in blood flow or interstitial fluid [11]. A strong argument against the assumption that MRI signal changes capture cortical neurogenesis comes from a recent *post mortem* study measuring the integration of (14)C, generated by nuclear bomb tests during the Cold War with DNA. This was used to establish the age of neurons in the major areas of the human cerebral neocortex and provided evidence, that neocortical neurogenesis may be restricted to the developmental period [12]. However, the contra argument is supported by the assumption that newly generated cells can migrate to distant anatomical sites [13]. Finally, a recent study by Pereira et al. demonstrating *in vivo* correlates of exercise-induced neurogenesis in the hippocampus confirms the theoretical possibility of angiogenesis underlying plasticity processes [14]. Further work is needed to clarify whether vascular changes due to increased cerebral blood volume and/or cerebral blood flow may have additional effects to the observed changes [15].

Independent of the precise histological nature of these structural alterations, our results support structural forms of neuroplasticity to be important in processing the information in dynamic networks according to novel informational demands [16]. Interestingly, neither performance (minutes endurance juggling) nor exercise (hours per day) was able to predict structural changes in the occipito-temporal cortex.

Importantly, the ability to initially learn a three-ball cascade juggling task is correlated with an increase in gray matter, whereas further improvement of the skill over time due to training does not seem to alter brain structure. Animal experiments suggest that learning is associated with synaptogenesis and glial hypertrophy, whereas a simple increase in motor activity is “only” related to angiogenesis [17]; [18].

As a general pattern, the increase in gray matter in all regions (Figure 1) is only detectable during constant training of the visual-motor skill and recedes when exercise is stopped, although the participants were still able to juggle. We suggest that the qualitative change (i.e. learning of a new task) is more critical for the brain to change its structure than simple training of this task once learned; however, when we detect such a change in brain structure, it may well be a combination of both. In the process of learning, it is a normative characteristic of the nervous system to change to be able to encode and appropriately implement new knowledge [19]. Further studies need to address the question whether the skill as such or whether exercising this skill is more important for functional and structural adaptations of the brain.

In addition to the gray matter change in the temporal area of the visual cortex, we found a change of brain gray matter which followed the same time pattern (increase during exercise and receding when exercise stopped) bilaterally in the frontal and temporal lobes and the cingulate cortex. Because this finding was not reported in our previous study [1] and because this finding did not survive the correction for multiple comparisons, these data have to be viewed with caution and may be unspecific. One possible reason why we detected these areas in the present study as compared to the study in 2004 may be the higher sensitivity of a higher field strength (3 Tesla vs. 1.5 Tesla) and/or of a larger group size in the present study (n = 20 vs. n = 12 in the former study) and an improved estimation of the mean and variance due to a higher number of repeated measures.

One of the unsolved obstacles of voxel based morphometry is the fact, that MR morphometry studies done at different research centers are almost impossible to compare due to scanner- and site- specific properties [5]. Therefore, multicenter studies are currently only feasible with significant limitations. The present study is the first to include data from two different cohorts scanned on two different scanners and even different field strengths.

It is an intriguing question why our brains do not expand over time, if we assume that that there is an increase in gray matter that is sustained with learning and/or practicing a skill. The most intuitive answer is, that the alterations are not sustained but that, once the learning process is over and the functional networks sufficient for the new task, the gray-matter changes reverse to their original size. However, given that such changes may last for at least 3 months without further exercising [19], we suggest that these regionally restricted changes are rather sublte and will not change the net-size or weight of the brain. It has also to be pointed out, that an increase in gray matter volume (i.e. a change of the classification of individual voxels from white to gray matter) will prompt an inverse effect (i.e. regionally loss in white matter volume) in adjacent white matter. The major future challenge is to understand the behavioural consequences and cellular mechanisms underlying training-induced neuroanatomic plasticity.
